# The added value of the selective SuperPolymyxin™ medium in detecting rectal carriage of Gram-negative bacteria with acquired colistin resistance in intensive care unit patients receiving selective digestive decontamination

**DOI:** 10.1007/s10096-019-03718-5

**Published:** 2019-11-06

**Authors:** Denise van Hout, Axel B. Janssen, Rob J. Rentenaar, Judith P.M. Vlooswijk, C.H. Edwin Boel, Marc J.M. Bonten

**Affiliations:** 1 Julius Center for Health Sciences and Primary Care, University Medical Center Utrecht, University Utrecht, Huispost nr. STR 6.131, P.O. Box 85500, 3508 GA, Utrecht, the Netherlands; 2 Department of Medical Microbiology, University Medical Center Utrecht, University Utrecht, Utrecht, the Netherlands

**Keywords:** Colistin resistance, Surveillance, Selective digestive decontamination, Selective medium, Colonization

## Abstract

The objective of this study was to determine the value of using SuperPolymyxin™ selective medium (ELITech Group, Puteaux, France) in addition to conventional non-selective inoculation methods in the detection of acquired colistin resistance in a Dutch intensive care unit (ICU) that routinely uses selective decontamination of the digestive tract (SDD). We performed a cross-sectional study with prospective data collection in a tertiary-care ICU. All consecutive surveillance rectal swabs of ICU-patients receiving SDD were included and cultured in an observer-blinded approach using (1) a conventional culture method using non-selective media and (2) SuperPolymyxin™ selective medium. MIC values for colistin of non-intrinsically colistin-resistant Gram-negative isolates were determined with broth microdilution (BMD) using Sensititre™ and colistin resistance was confirmed using BMD according to EUCAST guidelines. One thousand one hundred five rectal swabs of 428 unique ICU-patients were inoculated using both culture methods, yielding 346 and 84 Gram-negative isolates for BMD testing with the conventional method and SuperPolymyxin™ medium, of which 308 and 80 underwent BMD, respectively. The number of identified rectal carriers of isolates with acquired colistin resistance was 3 (0.7%) for the conventional method, 4 (0.9%) for SuperPolymyxin™, and 5 (1.2%) for both methods combined. The number of isolates with acquired colistin resistance was 4 (1.0%) for the conventional method, 8 (2.1%) for SuperPolymyxin™ and 9 (2.3%) for both methods combined. In a surveillance setting of low prevalence of acquired colistin resistance in patients that receive SDD in a Dutch tertiary-care ICU, SuperPolymyxin™ had a higher diagnostic yield than conventional inoculation methods, but the combination of both had the highest diagnostic yield.

**Electronic supplementary material:**

The online version of this article (10.1007/s10096-019-03718-5) contains supplementary material, which is available to authorized users.

## Introduction

Selective digestive decontamination (SDD) is a preventive antibiotic regimen that contains, among others, colistin as one of the topical components. SDD has been shown to reduce intensive care unit (ICU)-acquired infections and mortality in settings with low levels of antimicrobial resistance and is therefore standard of care in the Netherlands for ICU-patients [1, 2]. Colistin is increasingly regarded as a last-resort antibiotic against infections with multidrug-resistant Gram-negative bacteria (GNB) [3]. Dissemination of colistin resistance among GNB already resistant to other classes of antibiotics could potentially limit treatment options for patients infected with multidrug-resistant GNB, emphasizing the importance of optimizing surveillance and laboratory detection of colistin resistance.

Testing for phenotypic colistin-susceptibility however, is problematic [4–8]. A 2016 joint CLSI-EUCAST Working Group recommended that only broth microdilution (BMD) methods be used for testing of colistin susceptibility [9]. However, BMD requires manual preparation leading to potential errors, is labour-intensive and is difficult to implement in many routine clinical microbiology laboratories. Previous studies have suggested that the use of the commercially available SuperPolymyxin™ selective medium (ELITech Group, Puteaux, France) may improve the detection of colistin-resistant GNB in surveillance samples [10, 11].

The aim of the current study was to determine the added value of using SuperPolymyxin™ in addition to the conventional screening method with non-selective media in the detection of rectal carriage with acquired colistin-resistant GNB in a Dutch tertiary-care ICU that routinely uses SDD.

## Materials and methods

### Study design

A cross-sectional study with prospective data collection was performed from 9 July 2018 until 24 January 2019 in a 40-bed ICU of a tertiary care hospital in the Netherlands (University Medical Center Utrecht, Utrecht). All consecutive rectal swabs of ICU-patients taken during routine SDD surveillance were included and were taken at ICU-admission and twice weekly thereafter until ICU-discharge. Swabs were excluded in case of missing inoculation in either method. Ethical approval of patients was not deemed applicable because this was a laboratory quality improvement study and only anonymized medical microbiology data were used.

### Use of conventional non-selective media and the SuperPolymyxin™ medium

Rectal swabs were first inoculated on non-selective media (conventional method): tryptic soy, 5% sheep blood agar (BA, BD254087, Becton Dickinson, Erembodegem, Belgium), secondly on non-selective MacConkey agar (McC, BD257286) and thirdly on Malt extract agar (MEA, in-house manufactured). Fourthly, the swab was inoculated on the selective SuperPolymyxin™ medium. Plates were incubated at 37 °C for 48 h either in 5% CO_2_ (BA), or in ambient air (McC, MEA, SuperPolymyxin™). All plates were visually examined after 24 and 48 h of incubation. Technicians that visually inspected growth on the conventional method were blinded for results of the SuperPolymyxin™ medium, and vice versa. All different colony morphologies that were suspected of being GNB were subjected to species identification by MALDI-TOF MS (Bruker, Bremen, Germany) and all Gram-negative isolates were stored at − 80 °C.

### Colistin broth microdilution

MIC determination was performed on all Gram-negative isolates of species that are not intrinsically resistant to colistin using Sensititre™ FRCOL plates (Thermo Fisher Scientific, Wesel, Germany) according to the manufacturer’s instructions. *E. coli* ATCC25922 and *E. coli* NCTC13846 were included as control strains daily. Colistin MICs were interpreted according to EUCAST 2019 guidelines [12]. Colistin resistance of isolates that were tested colistin-resistant with Sensititre™ was confirmed using a broth microdilution method in line with EUCAST guidelines [9, 13]. *Stenotrophomonas maltophilia* and *Achromobacter xylosoxidans* were excluded from analyses because of missing EUCAST and CLSI susceptibility breakpoints for colistin.

### *Mcr*-gene detection

Genomic sequences of the isolates that tested colistin-resistant with BMD were subjected to screening for *mcr-*genes using ResFinder 3.2 [14].

### Statistical analyses

Contingency tables were made for the conventional method compared to SuperPolymyxin™ in the detection of ICU-patients with a rectal swab positive for ≥ 1 isolate that exhibited acquired colistin resistance at any time point during ICU-stay and rectal swabs positive for ≥ 1 isolate that exhibited acquired colistin resistance. Acquired colistin resistance was defined as colistin resistance determined with BMD in species that are usually susceptible to colistin. Isolates from a single rectal swab with different colony morphologies on the SuperPolymyxin™ plate but belonging to identical species and with similar colistin MICs were counted only once. The number of isolates that grew on SuperPolymyxin™ but were colistin-susceptible with BMD using Sensititre™ (i.e. false-resistant result) was reported, as well as the number of colistin-resistant isolates that was found in the conventional method but did not grow on SuperPolymyxin™ medium (i.e. false-susceptible result).

The value of SuperPolymyxin™ as a screening method for routine colistin-susceptibility testing was examined by comparing the use of SuperPolymyxin™ with implementation of routine colistin BMD using Sensititre™. Three scenarios were compared: (1) implementation of routine colistin Sensititre™ BMD for all Gram-negative isolates detected in the conventional (non-selective) method, (2) addition of SuperPolymyxin™ to the current laboratory pipeline and performing colistin Sensititre™ BMD on all isolates detected in either the conventional method or SuperPolymyxin™ and (3) addition of SuperPolymyxin™ to the current pipeline and only performing colistin Sensititre™ BMD on isolates detected through SuperPolymyxin™ (i.e. using SuperPolymyxin™ as a screening medium). We calculated maximum costs per SuperPolymyxin™ plate for the use of SuperPolymyxin™ as a screening medium to be under the costs of performing colistin BMD using Sensititre™ on all isolates detected with the conventional method.

This was a pragmatic study without a formal sample size calculation; the aim was to include 1000 rectal swabs. All analyses were performed with Statistical Package for Social Sciences V.25.0 (SPSS, Chicago, Illinois, USA).

## Results

### ICU-patients, rectal swabs and Gram-negative isolates for colistin broth microdilution

**Fig. 1:**
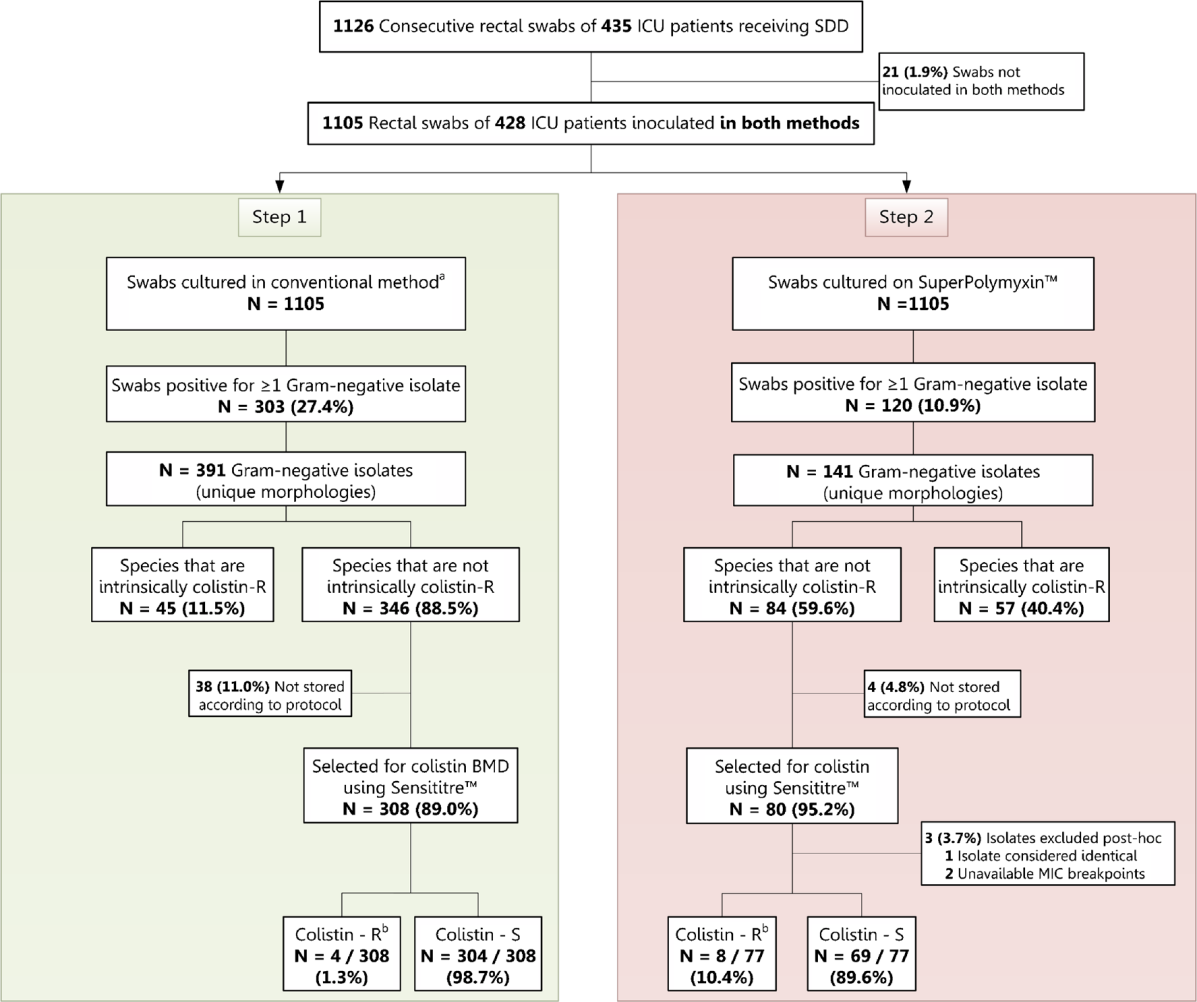
Study flowchart. *BMD*, broth microdilution; *ICU*, intensive care unit; *McC*, MacConkey agar; *MEA*, malt extract agar; *R*, resistant; *S*, susceptible; *SDD*, selective digestive decontamination. ^a^ The conventional method consisted of inoculation on non-selective Blood (BA) and MacConkey (McC) agar and Malt extract agar. Only BA and McC were used for isolation of Gram-negative isolates. ^b^ Acquired colistin resistance was confirmed using a broth microdilution method in line with EUCAST guidelines [9, 13] (see “Methods” section).

A total of 1105 rectal swabs of 428 unique ICU-patients were included (Fig. 1). The conventional method and SuperPolymyxin™ medium yielded 308 and 77 Gram-negative isolates that were tested with colistin BMD using Sensititre™ and were included in further analyses, respectively (Fig. 1).

### Diagnostic yield

The number of carriers and positive rectal swabs was highest when combining the results of both methods (Tables 1 and 2). Colistin susceptibility pattern was reclassified from resistant into susceptible in 2 isolates after BMD with cation-adjusted Mueller Hinton broth (1 *E. coli* and 1 *P. aeruginosa*). The number of isolates with acquired colistin resistance was 4 (4/385 = 1.0%) with the conventional method, 8 (8/385 = 2.1%) with SuperPolymyxin™ medium and 9 (9/385 = 2.3%) with both methods combined (Tables 1 and 3). In total, 373 colistin-susceptible isolates were identified, of which 69 (18.5%, 95% CI 14.9–22.8%) grew on the SuperPolymyxin™ medium. Of the 9 unique isolates with acquired colistin resistance, 1 (11.1%, 95% CI 2.0–43.5%) did not exhibit growth on SuperPolymyxin™ (Table 3 and Online Resource 1).

**Table 1:** Diagnostic yield per inoculation method

	Conventional method^a^	SuperPolymyxin™
ICU patients	*N* = 428 (%)	*N* = 428 (%)
Rectal carriers of GNB with acq. colistin resistance	3 (0.7)	4 (0.9)
Rectal swabs	*N* = 1105 (%)	*N* = 1105 (%)
Rectal swabs with ≥ 1 GNB with acq. colistin resistance	4 (0.4)	8 (0.7)
Colistin-resistant isolates		
Species intrinsically colistin-resistant	*N* = 45 (%)	*N* = 57 (%)
*Proteus mirabilis*	23 (51.1)	19 (33.3)
*Morganella morganii*	7 (15.6)	16 (28.1)
*Serratia marcescens*	8 (17.8)	11 (19.3)
*Providencia rettgeri*	4 (8.9)	3 (5.2)
*Hafnia alvei*	1 (2.2)	4 (7.0)
*Ochrobactrum intermedium*	1 (2.2)	2 (3.5)
*Proteus vulgaris*	1 (2.2)	1 (1.8)
*Providencia species*	–	1 (1.8)
Species non-intrinsically colistin-resistant	*N* = 4 (%)	*N* = 8 (%)
*Escherichia coli*	2 (50.0)	6 (75.0)
*Klebsiella aerogenes*	1 (25.0)	2 (25.0)
*Enterobacter asburiae*	1 (25.0)	–

A*cq*, acquired; *GNB*, Gram-negative bacteria; *ICU*, intensive care unit ^a^The conventional method consisted of inoculation on non-selective Blood (BA) and MacConkey (McC) agar and Malt extract agar. Only BA and McC were used for isolation of Gram-negative isolates.

**Table 2:** Comparison of the conventional method^a^ and SuperPolymyxin™ medium in the detection of rectal carriers of Gram-negative isolates that exhibited acquired colistin resistance and rectal swabs positive for Gram-negative isolates that exhibited acquired colistin resistance

		SuperPolymyxin™		
Conventional method^a^	**A***.* ICU patients	Carrier	Non-carrier	
	Carrier	*2*	*1*	3
	Non-carrier	*2*	*423*	425
		4	424	
	**B**. Rectal swabs	Positive	Negative	
	Positive	*3*	*1*	4
	Negative	*5*	*1096*	1101
		8	1097	

^a^The conventional method consisted of inoculation on non-selective blood (BA) and MacConkey (McC) agar and Malt extract agar. Only BA and McC were used for isolation of Gram-negative isolates.

**A.** Number of detected rectal carriers and non-carriers of ≥ 1 non-intrinsically colistin-resistant isolate with acquired colistin resistance. **B.** Number of swabs detected that were positive or negative for ≥ 1 non-intrinsically colistin-resistant isolate with acquired colistin resistance.

**Table 3:** Culture results from the conventional method and SuperPolymyxin™ medium for all rectal swabs with growth of acquired colistin-resistant Gram-negative isolates

**PT**	Swab	Date	Growth on conventional method^a^			Growth on SuperPolymyxin™		
			Species	Colistin MIC^b^ (μg/mL)	Colistin profile	Species	Colistin MIC^b^ (μg/mL)	Colistin profile
27	282	30 August 2018	*E. coli*	0.5	S	–	–	–
			–	–	–	**E. coli**^c^	**8**	**R**
			*K. pneumoniae*	1	S	*K. pneumoniae*	1	S
31	643	01 November 2018	*E. coli*	16	R	*E. coli*	16	R
	661	05 November 2018	*E. coli*	NA	NA	**E. coli**	**16**	**R**
	681	08 November 2018	*E. coli*	NA	NA	**E. coli**	**16**	**R**
	722	14 November 2018	*E. coli*	16	R	*E. coli*	16	R
37	146	06 August 2018	*K. aerogenes*	0.5	S	–	–	–
			–	–	–	**K. aerogenes**	**32**	**R**
	177	09 August 2019	*K. aerogenes*	32	R	*K. aerogenes*	32	R
			*–*	–	–	*M. morganii*	–	R
307	331	07 September 2018	**E. asburiae**	**> 128**	**R**	*–*	–	–
			*E. coli*	0.5	S	*–*	–	–
311	636	01 November 2018	*E. coli*	NA	NA	**E. coli**	**4**	**R**

*MIC*, minimum inhibitory concentration; *NA*, not available (i.e. not stored); *PT*, patient; *S*, sensitive; *R*, resistant

Total growth of all rectal swabs with growth of a Gram-negative isolate with acquired colistin resistance is presented (including intrinsically colistin-resistant and/or colistin-sensitive Gram-negative isolates, if these grew on the rectal swabs). Data in bold indicates discordant growth of isolates with acquired colistin resistance between the two methods.

^a^The conventional method consisted of inoculation on non-selective Blood (BA) and MacConkey (McC) agar and Malt extract agar. Only BA and McC were used for isolation of Gram-negative isolates.

^b^Colistin MICs by using the commercial Sensititre™ broth microdilution method. Presence of acquired colistin resistance was confirmed using a broth microdilution method in line with EUCAST guidelines (see “Methods” section).

^c^Tested positive for *mcr*-1.

### Characteristics of isolates with acquired colistin resistance

Colistin MICs of isolates with acquired colistin resistance ranged from 4 to > 128 μg/mL (Table 3). One *E. coli* isolate tested positive for *mcr-1*; this isolate was identified with SuperPolymyxin™ and had a colistin MIC of 8 μg/mL. No other *mcr*-genes were identified.

### Added value of SuperPolymyxin™ medium as screening method

**Fig. 2:**
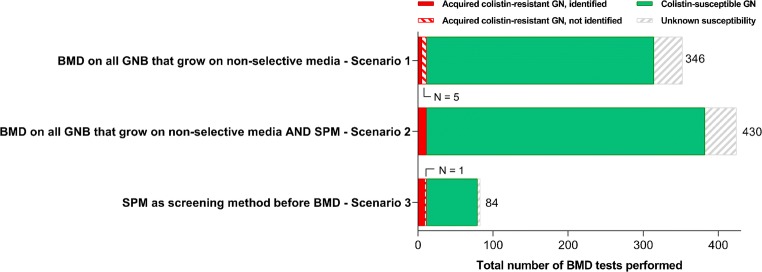
Different scenarios for implementing colistin BMD using Sensititre™ in the current laboratory pipeline. *BMD*, broth microdilution; *GN*, Gram-negative isolates; *SPM*, SuperPolymyxin™ medium

A strategy of BMD testing of all isolates that grew on non-selective media and/or the SuperPolymyxin™ medium (scenario 2, Fig. 2) would have the highest diagnostic yield and would require 430 Sensititre™ BMD tests. A strategy in which only isolates identified with SuperPolymyxin™ would undergo Sensititre™ BMD would require 84 BMD tests, a reduction of 75.7% (1–84/346) (scenario 3 versus 1), at the cost of 1 missed isolate with acquired colistin resistance. Not using SuperPolymyxin™ medium (scenario 1) would require 346 Sensititre™ BMD tests (19.5% less than when also using SuperPolymyxin™ medium) and would have let to 5 missed isolates with acquired colistin resistance (including 1 *mcr-1* positive *E. coli*). Considering the direct costs of Sensititre™ BMD (in our laboratory being €21.45 per test), addition of SuperPolymyxin™ to the conventional inoculation methods as a screening medium (scenario 3) would be cheaper than performing Sensititre™ BMD on all isolates that grew in the conventional method (scenario 1) if the costs of adding SuperPolymyxin™ would not exceed €5.09 per Sensititre™ BMD test (including laboratory technician time) (see Online Resource 2).

## Discussion

In this prospective study, embedded in routine surveillance of ICU-patients that receive SDD, the combined use of conventional inoculation methods and selective SuperPolymyxin™ medium had the highest diagnostic yields in detecting rectal carriers of isolates that exhibited acquired colistin resistance, rectal swabs positive for isolates that exhibited acquired colistin resistance and the total number of detected colistin-resistant Gram-negative isolates.

Previous studies that assessed the diagnostic performance and applicability of the commercial SuperPolymyxin™ medium in routine screening reported varying results [10, 15, 16]. In one study, rectal swabs were spiked with 94 well-characterized *Enterobacterales* (of which 53 with acquired colistin resistance), and sensitivity and specificity of the SuperPolymyxin medium™ were 86.8% (95% CI 74.0–94.0%) and 97.5% (95% CI 85.6–99.9%), respectively [16]. In another study, 100% (33/33) sensitivity and 90.3% (56/62) specificity of SuperPolymyxin™ were reported [10]. In the current study, we did not aim to determine sensitivity and specificity of the test. However, we did find one false-negative result of the SuperPolymyxin™ medium (11.1%, 95% CI 2.0–43.5%). A possible explanation could be that SuperPolymyxin™ was inoculated as the fourth medium, potentially leading to reduced bacterial loads on rectal swabs upon inoculation of SuperPolymyxin™. Our inoculation method might bias our results towards false-negative results of SuperPolymyxin™ in case of low density inocula. However, it is currently standard procedure to inoculate plates directly from rectal swabs, with a standard order from non-selective media to selective media. Thus, this is how SuperPolymyxin™ medium would be used in our routine practice, which was the main research aim of the current study.

Strengths of the current study were the prospective data collection and study design, which were embedded in our current routine laboratory pipeline of SDD surveillance. To decrease observer bias, all observers were blinded for the results of the alternative method. One of the study limitations was the number of isolates that were not stored according to protocol, as one of the technicians was not aware of the study instruction to store all Gram-negative isolates that grew on either method. Another limitation was that the total amount of identified isolates with acquired colistin resistance was relatively low. It is known that the performance of SuperPolymyxin™ is different for different Gram-negative species, so it is important to note that some important species were not encountered during our study period (i.e. *Salmonella* sp., *Acinetobacter baumannii*) [10]. This could have influenced the determination of error rates of the SuperPolymyxin™ medium (in unknown direction). Use of an enrichment broth might have increased diagnostic yield; however, this was not included as part of the current study. Lastly, this study was performed in a single-center ICU that routinely uses SDD, and results therefore may not be generalizable to all other clinical settings.

We tested a two-step approach, in which screening through SuperPolymyxin™ was followed by colistin BMD testing using Sensititre™. Naturally, it is important to consider both additional value and costs before implementing new diagnostic tools. The added value depends on the aim of colistin susceptibility testing (i.e. research, surveillance or direct patient care), the clinical impact of identifying colistin resistance and the impact of potential missed cases. In our setting with low prevalence of colistin resistance, total diagnostic yield will always be low. Still, given the resource-dense nature of colistin BMD testing, results of the current study support the use of colistin-selective media as a screening method in case of daily large numbers of screening samples, such as in our SDD surveillance setting. Future research could determine the value of SuperPolymyxin™ in other settings, for example in laboratories in which colistin BMD testing is already part of routine practice or in countries with higher prevalence of colistin resistance.

In conclusion, in a routine surveillance setting of ICU-patients that receive SDD, the combined use of non-selective media and selective SuperPolymyxin™ medium had the highest diagnostic yield in detecting Gram-negative isolates with acquired colistin resistance. However, overall prevalence of acquired colistin resistance was low.

## Electronic supplementary material

ESM 1 (DOCX 16 kb)

ESM 2 (PDF 145 kb)
