# Supplementary material for: The added value of the selective SuperPolymyxin™ medium in detecting rectal carriage of Gram-negative bacteria with acquired colistin resistance in intensive care unit patients receiving selective digestive decontamination
Source: Eur J Clin Microbiol Infect Dis. 2019 Nov 6;39(2):265–71. doi: 10.1007/s10096-019-03718-5 (PMC7010615; doi:10.1007/s10096-019-03718-5)
Supplement: Supplementary file 1 — (DOCX 16 kb) [file 10096_2019_3718_MOESM1_ESM.docx]

**SUPPLEMENTARY MATERIAL – FILE 2**

This is supplementary material to the manuscript:

The added value of the selective SuperPolymyxin™ medium in detecting rectal carriage of Gram-negative bacteria with acquired colistin resistance in intensive care unit patients receiving selective digestive decontamination
Denise van Hout*, Axel B. Janssen, Rob J. Rentenaar, Judith P.M. Vlooswijk, C.H. Edwin Boel, Marc J.M. Bonten

*E-mail corresponding author: D.vanHout-3@umcutrecht.nl

**Table 1S.** Performance of the SuperPolymyxin™ medium in the detection of Gram-negative isolates with acquired colistin resistance

|  |  | **Colistin resistance as determined with BMD**^a^ | | |
| --- | --- | --- | --- | --- |
|  |  | **+** | **-** | |
| **Growth on SuperPolymyxin™** | **+** | **8** | **69** | 77 |
|  | **-** | **1** | **304** | 305 |
|  |  | 9^b^ | 373 | 382 |
| BMD, broth microdilution  ^a^ BMD was only performed on Gram-negative isolates that are not intrinsically resistant to colistin and was performed by first using Sensititre™ on all isolates, followed by BMD using Mueller Hinton cation-adjusted broth for the isolates that were tested colistin resistant with Sensititre™.  ^b^ Unique Gram-negative isolates with acquired colistin resistance (i.e. the 3 colistin-resistant isolates that were detected in both methods were only counted once in this table) | | | | |
