# Supplementary material for: The added value of the selective SuperPolymyxin™ medium in detecting rectal carriage of Gram-negative bacteria with acquired colistin resistance in intensive care unit patients receiving selective digestive decontamination
Source: Eur J Clin Microbiol Infect Dis. 2019 Nov 6;39(2):265–71. doi: 10.1007/s10096-019-03718-5 (PMC7010615; doi:10.1007/s10096-019-03718-5)
Supplement: Supplementary file 2 — (PDF 145 kb) [file 10096_2019_3718_MOESM2_ESM.pdf]

Cost analysis different scenarios of implementing colistin broth microdilution

Supplementary material to: 'The added value of the selective SuperPolymyxin™ medium in detecting rectal carriage of Gram-negative bacteria with acquired colistin resistance in intensive care unit patients receiving selective digestive decontamination'

Date: 14 June 2019

Author: D. van Hout, e-mail: D.vanHout-3@umcutrecht.nl

Scenario 1: Implementation of routine colistin broth microdilution (BMD) on all Gram-negative isolates detected in the conventional inoculation method.

Scenario 2: Addition of SuperPolymyxin™ medium (SPM) to the current laboratory pipeline and performing colistin BMD on all isolates detected in either the conventional method or SuperPolymyxin™ medium.

Scenario 3: Addition of the SuperPolymyxin™ medium to the current laboratory pipeline and only performing colistin BMD on isolates detected through SuperPolymyxin™ (i.e. using SuperPolymyxin™ medium as a screening medium).

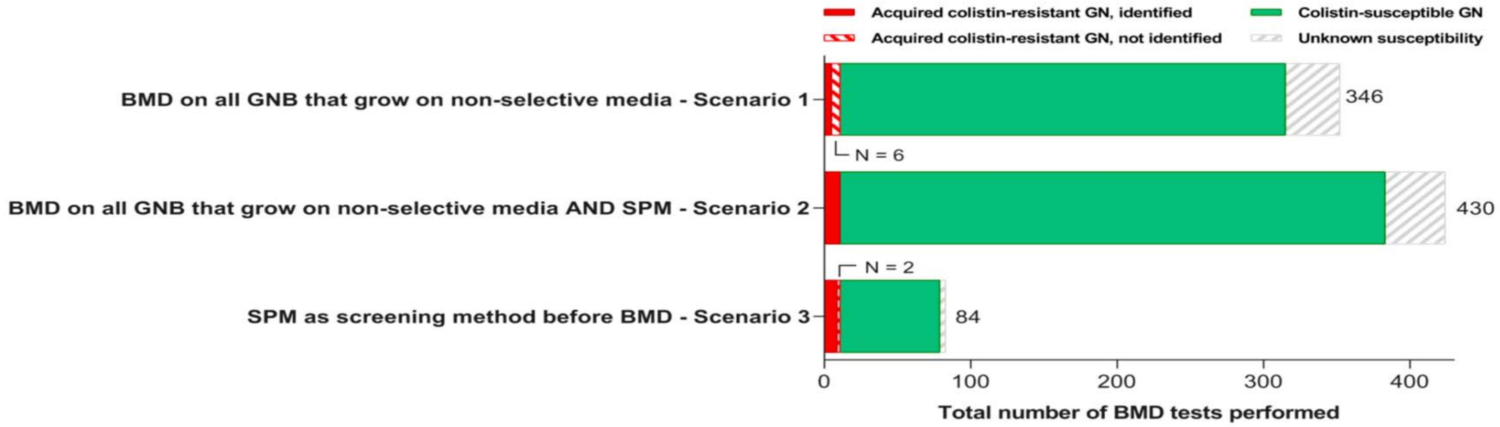

Notes:

1. Costs of conventional inoculation methods are considered the same in each scenario; because SuperPolymyxin would be added to the current pipeline and would never "replace' all conventional methods. Therefore, these costs are not taken in to account in the current cost analysis. **IMPORTANT:** Costs represented here should therefore NOT be interpreted as total absolute costs for the different scenarios.
2. The costs per colistin BMD test (21.45 euro) are based on internal UMCU calculations and are available from the corresponding author. These costs include material, lab technician time, overhead, etc.
3. The current cost analysis is based on the number of isolates that were found during the study period: 9-7-2018 until 24-1-2019 (approx 6.5 months).

	**Scenario 1**			**Scenario 2**			**Scenario 3**		
	N tests performed	Costs per test	Total costs	N tests performed	Costs per test	Total costs	N tests performed	Costs per test	Total costs
Costs SPM (hypothetical)	0	0	0	1105	€ 5,09	€ 5.619,90	1105	€ 5,09	€ 5.619,90
Costs BMD	346	€ 21,45	€ 7.421,70	430	€ 21,45	€ 9.223,50	84	€ 21,45	€ 1.801,80
**Total costs**			**€ 7.421,70**			**€ 14.843,40**			**€ 7.421,70**

Interpretation:

1. If the SPM medium would be 5.09 euro per plate, including lab technician time, Scenario 1 would be equal in costs as compared to Scenario 3.
2. If the SPM medium would be <5.09 euro per plate (including material, lab technician time, etc.), then Scenario 3 would be cheaper as compared to Scenario 1.
3. Scenario 2 is the most expensive scenario.
